# Demonstration of feasibility and technique of transesophageal endoscopic epicardial access in a porcine model

**DOI:** 10.1016/j.igie.2023.10.008

**Published:** 2023-10-20

**Authors:** Zachary N. Weitzner, Steven Cha, Ronald Challita, Olujimi Ajijola, Shumpei Mori, Kalyanam Shivkumar, Erik Dutson, Alireza Sedarat

**Affiliations:** David Geffen School of Medicine, University of California Los Angeles, Los Angeles, California, USA; Current affiliation: David Geffen School of Medicine, University of California Los Angeles, Los Angeles, California, USA

## Abstract

**Background and aims:**

Peroral endoscopic myotomy uses a submucosal tunnel that staggers the esophageal mucosotomy and myotomy, allowing minimally invasive interventions in the esophagus to treat achalasia. Our goal was to assess the feasibility of this method to access the mediastinum to perform endoscopic epicardial access and pericardial window in a porcine model.

**Methods:**

Experiments were conducted to assess the feasibility of this procedure. Commonly available ERCP and EUS instruments were used to show epicardial access under both direct transesophageal mediastinoscopic visualization and EUS guidance.

**Results:**

In experiments, we successfully placed catheters in the pericardial space without hemodynamic instability or adverse events. A pericardial window was performed in other experiments, draining the instilled simulated pericardial effusion.

**Conclusion:**

Transesophageal epicardial access and pericardial window seemed to be feasible and well tolerated in a porcine model, indicating the possibility of expanding mediastinal procedures conducted with endoscopy in the future.

As the fields of interventional EUS, third space endoscopy, and surgical endoscopy have developed, therapeutic endoscopists have become increasingly capable of performing complex interventions and accessing a growing number of extraluminal spaces. Experience with peroral endoscopic myotomy (POEM) shows the tolerability of staggered mucosotomies and myotomies in the esophagus, as well as the rapid reabsorption of carbon dioxide insufflation from the mediastinum.^1^ This suggests the potential feasibility of endoscopic approaches to spaces outside of the esophageal lumen as an alternative to laparoscopic or thoracoscopic approaches.

Our team has shown the survivability of a transesophageal approach to vagotomy in a porcine model.^2^ We sought to assess the feasibility of obtaining epicardial access via an endoscopic approach in a porcine model to guide potential alternatives to existing clinical applications of percutaneous, transatrial, or trans-sinus access in the future. We conducted 2 experimental protocols for epicardial access: one using a transesophageal natural orifice transluminal endoscopic surgery (NOTES) approach and a second using transesophageal EUS.

In the absence of pathology, the pericardial space is a potential space between the outer parietal pericardium and inner visceral pericardium with only 15 to 50 mL of fluid.^3^ Percutaneous epicardial access is obtained for access to the surface of the epicardium for interventions to protect the esophagus from thermal injury during electrophysiological ablation of arrhythmias. However, percutaneous epicardial access can be difficult due to the small depth of this space.^4^

Traditionally, epicardial access is obtained percutaneously with the Sosa technique, using a subxiphoid percutaneous needle under fluoroscopic guidance to enable a catheter to be placed via the Seldinger technique.^5^ However, the incidence of adverse events is relatively high.^4^, ^5^, ^6^ Inadvertent right ventricular puncture reportedly occurs in 4.5% to 17%, and injury to coronary vessels has been described, requiring operative intervention in 1.7% of epicardial access attempts.^4^^,^^5^ In addition, pericardial window carries a risk of perioperative death of approximately 7%, with a >10-day average length of stay and large narcotic requirements, although typically performed in the setting of cardiac tamponade.^7^ Because pericardial expansion is sought to prevent injury to the esophagus in clinical practice, we hypothesized that the feasibility of access to the pericardial space from the esophagus could be possible.

## Methods

This study was approved by our institution’s Animal Review Committee and conducted in accordance with U.S. Department of Agriculture Covered Species requirements. Experiments were performed on 4 male Yorkshire pigs weighing between 38.0 kg and 46.9 kg. Four experiments were conducted in total, 1 necropsy and 1 terminal procedure for each of the 2 endoscopic approach techniques. Pigs were anesthetized according to institutional protocol. Blood pressure, electrocardiogram, oxygen saturation, and temperature were continually monitored.

### Directly visualized epicardial access and endoscopic pericardial window via NOTES mediastinal dissection

A standard gastroscope (EG-760; Fujifilm, Tokyo, Japan) was used to inspect the esophagus and stomach. The gastroesophageal junction was 61 cm from the incisors and the upper esophageal sphincter at 21 cm. In a technique similar to POEM, a 23-gauge Interject needle (Boston Scientific, Marlborough, Mass, USA) was used to inject methylene blue dye and saline to expand the submucosal space, separating the mucosa and muscularis propria and creating a dissection plane. An ORISE ProKnife (Boston Scientific) was used to create a 1.5- to 2-cm incision in the mucosa, allowing the endoscope to tunnel caudally in the submucosal plane. The point of esophageal entry was selected strategically for a favorable approach to the heart based on endoscopic and fluoroscopic landmarks.

The submucosal tunnel staggers the openings in the mucosa and muscularis propria, preventing full-thickness esophageal defects, and allows for closure without mediastinitis. This approach follows principles established in the early NOTES experience termed submucosal endoscopy with mucosal flap.^8^ After tunneling 4 cm caudally, another 2-cm incision was made in the muscularis, allowing the endoscope to pass through the esophageal wall into the mediastinum.

**Figure 1:**
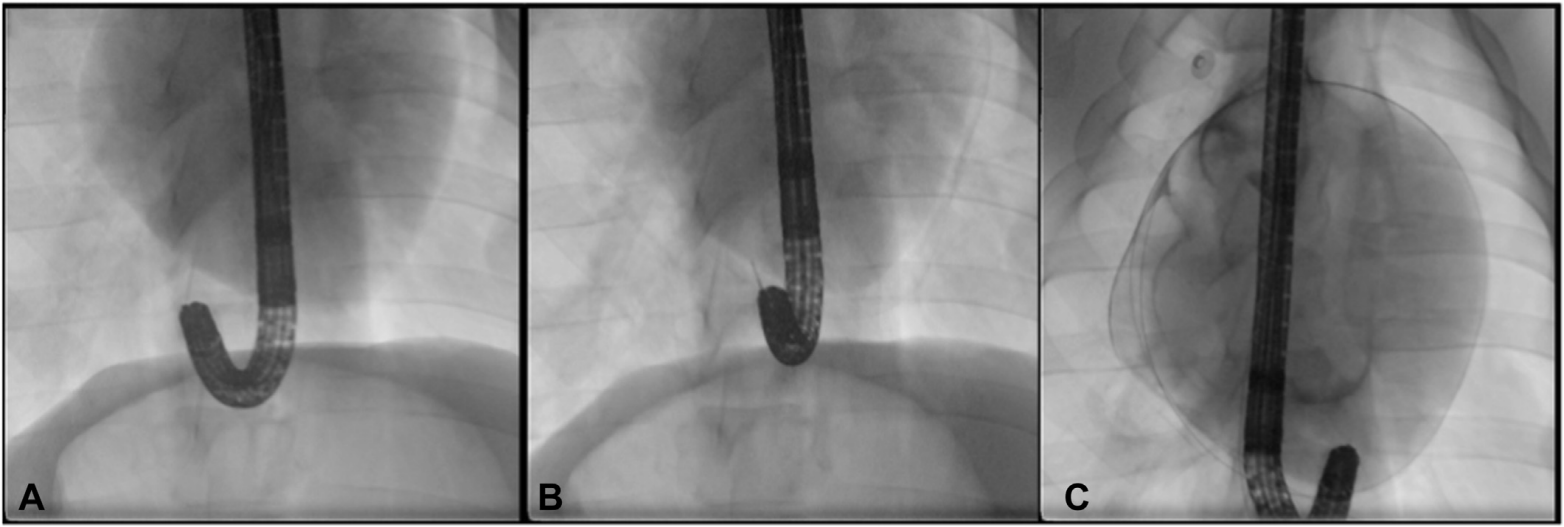
(**A**) Retroflexion was used in the mediastinum to aim cephalad for pericardial intervention. (**B**) Entry into the pericardium was confirmed with fluoroscopy and the pericardium insufflated with carbon dioxide. (**C**) A wire was then inserted into the pericardium, obtaining access, and a catheter subsequently inserted.

After entering the mediastinum, the scope was retroflexed and fluoroscopy was used to guide endoscopic dissection toward the right ventricle (Fig. 1). Dissection with ProKnife electrocautery was used to pass through thin avascular mediastinal areolar tissue. This dissection is relatively straightforward, with no significant bleeding or thick tissue. Instillation of blue dye and saline assists with identification of avascular planes.

**Figure 2:**
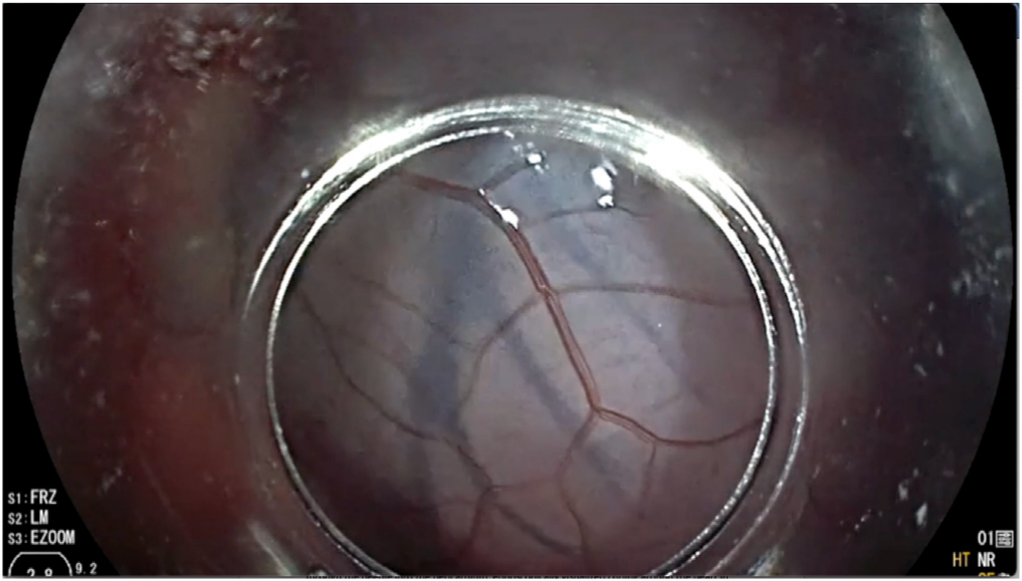
Visualization of the epicardial vessels through the pericardium.

**Figure 3:**
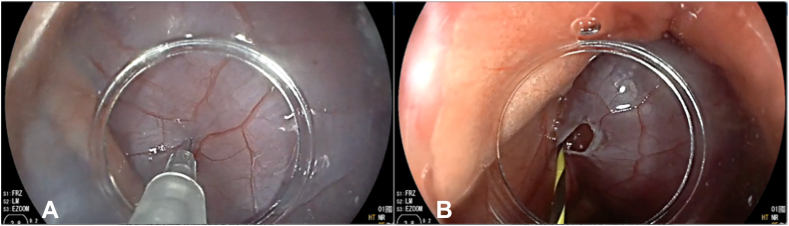
Insufflation of the pericardial space (**A**) and insertion of a wire into the pericardial space (**B**).

Upon visualization of the heart, a location away from epicardial vessels was then identified for epicardial access (Fig. 2). A 23-gauge Interject needle was used to puncture the parietal pericardium at an oblique angle, with care taken to avoid ventricular puncture (Fig. 3). Proper positioning of the needle was confirmed with injection of iohexol fluoroscopically visualized filling the pericardial space. Thie technique is similar to injections commonly performed for submucosal endoscopy procedures but made more challenging due to beating of the heart.

**Figure 4:**
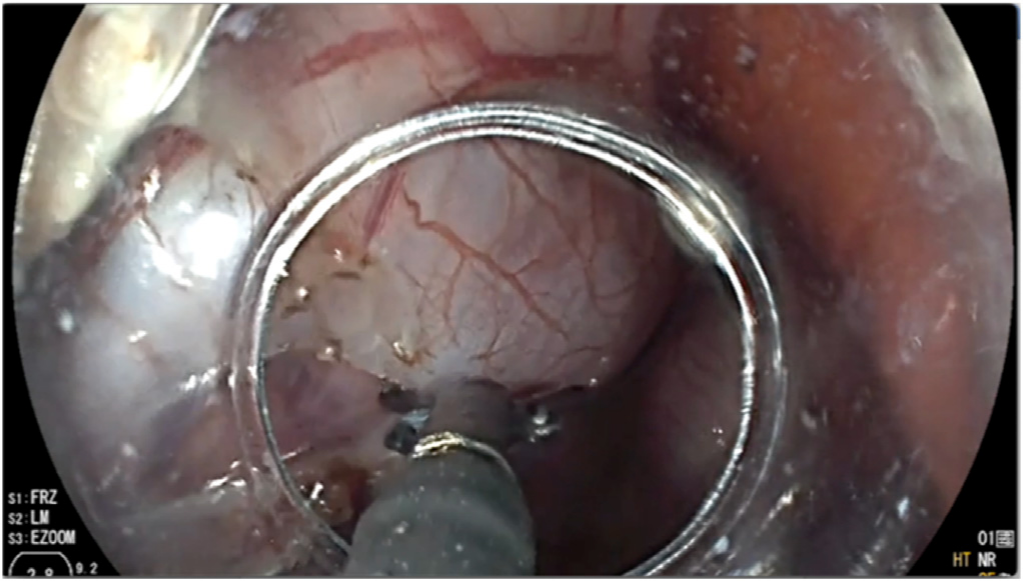
Pericardial window made with endoscopic cutters to facilitate pericardial drainage.

The space was insufflated with carbon dioxide, showing expansion of the pericardium fluoroscopically and an adequate simulated target for potential percutaneous access that was the goal of the experiment. The needle puncture site was then incised and slightly expanded with an electrosurgical knife, which was done safely due to the gas-expanded space. Through the pericardiotomy, a 0.025-inch Jagwire guidewire (Boston Scientific) was passed under fluoroscopic and endoscopic guidance the pericardial space (Fig. 2). A 5.5F Tandem XL Triple-Lumen ERCP Cannula (Boston Scientific) was passed over the wire into the pericardial space, achieving catheter epicardial access. Next, an approximately 2-cm pericardial window was created, opening the pericardium and pleural spaces, allowing free drainage of pericardial fluid, confirmed fluoroscopically by emptying of the previously instilled pericardial contrast (Fig. 4).

**Figure 5:**
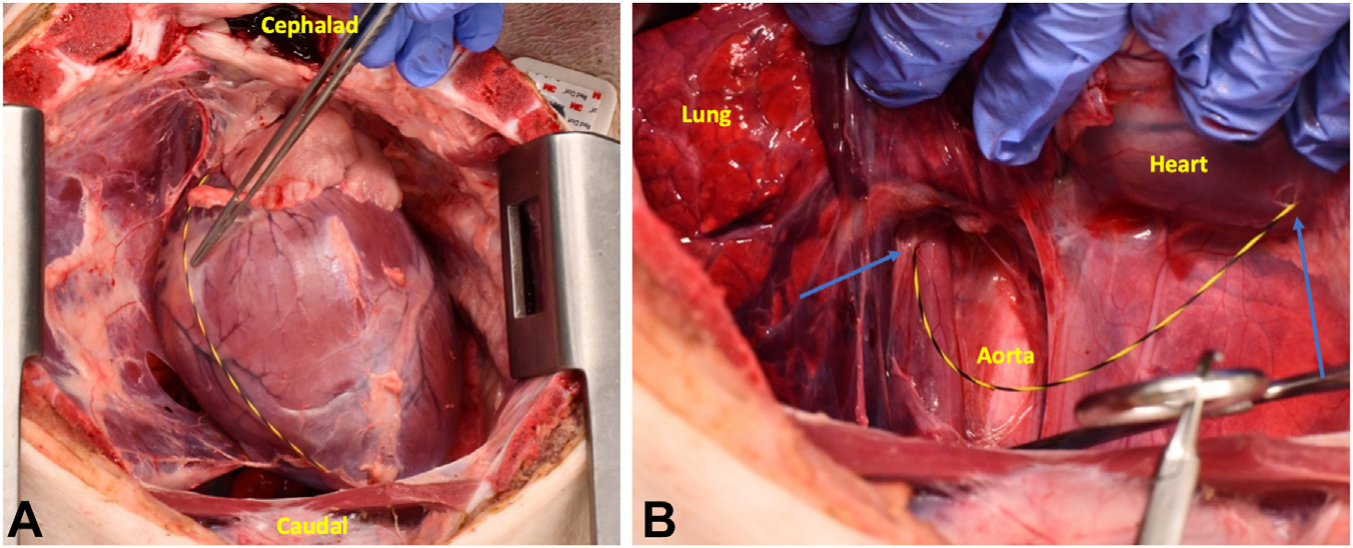
Wire in the pericardial space (**A**) and exiting the esophagus and entering the pericardium (**B**). *Blue arrow* on the left marks the esophagus with exit site of scope to the mediastinum, and the *blue arrow* on the right marks pericardiotomy and entrance of the wire into the pericardial space seen in left image.

Gas and fluid were suctioned as the endoscope was exchanged out, the wire was left in place in the pericardium, and the procedure was completed. Total procedure time was approximately 90 minutes via this NOTES approach. After procedure termination, a necropsy was performed. The wire was visualized leaving the esophageal wall, passing through the inferior mediastinum, and entering the pericardium (Fig. 5, Video 1, available online at www.igiejournal.org).

### EUS-guided direct trans-esophageal epicardial access

**Figure 6:**
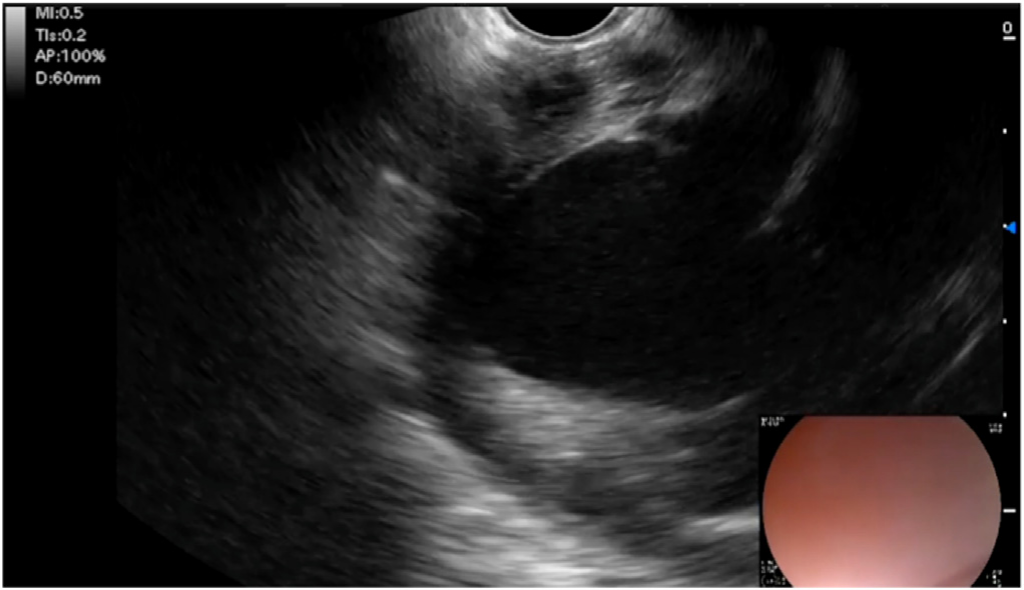
EUS of the left ventricle from the esophagus.

A linear echoendoscope (EG-580UT; Fujifilm) was used to identify an area of the heart adjacent to the esophagus, where the left ventricle was in proximity to the anterior esophageal wall (Fig. 6).

**Figure 7:**
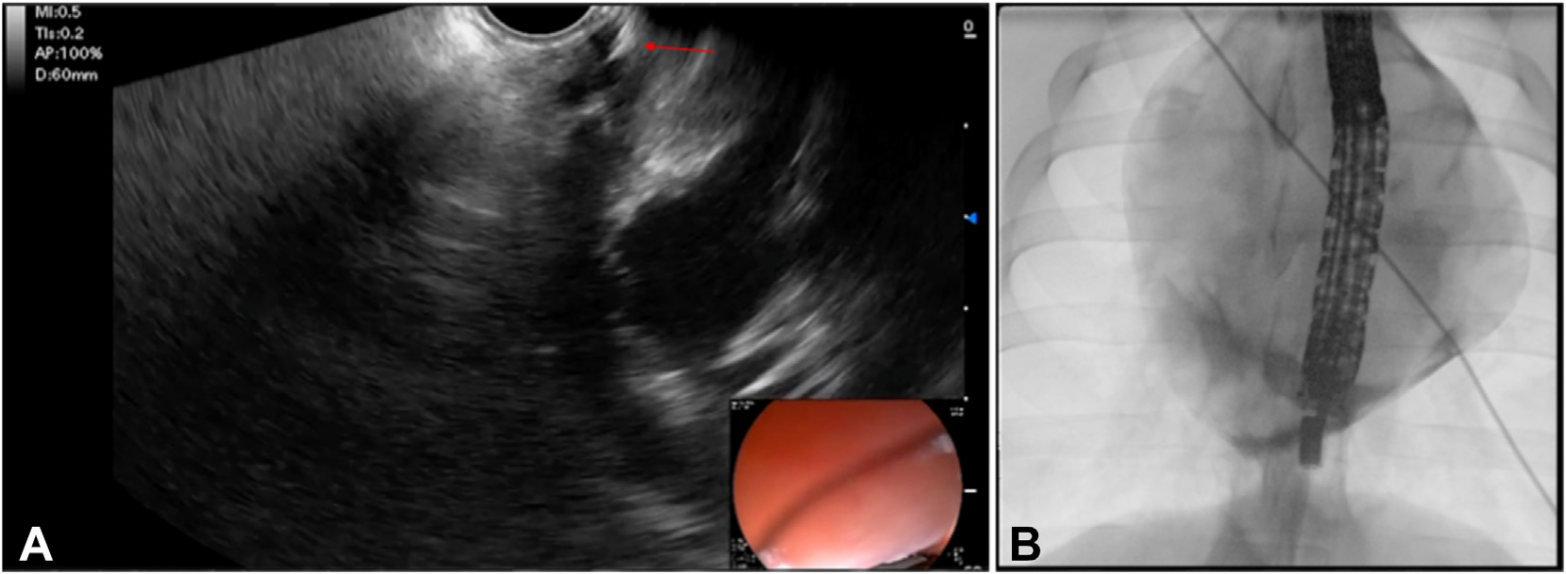
The needle (*red arrow*) was carefully passed into the pericardial space (**A**). Positioning was confirmed with fluoroscopy (**B**); contrast can be seen pooling in the inferior pericardium.

Doppler was used to identify an area free of large epicardial vessels. A 19-gauge fine aspiration needle (Expect; Boston Scientific) was then advanced into the pericardial space, using iohexol contrast injection and fluoroscopy to confirm positioning (Fig. 7).

**Figure 8:**
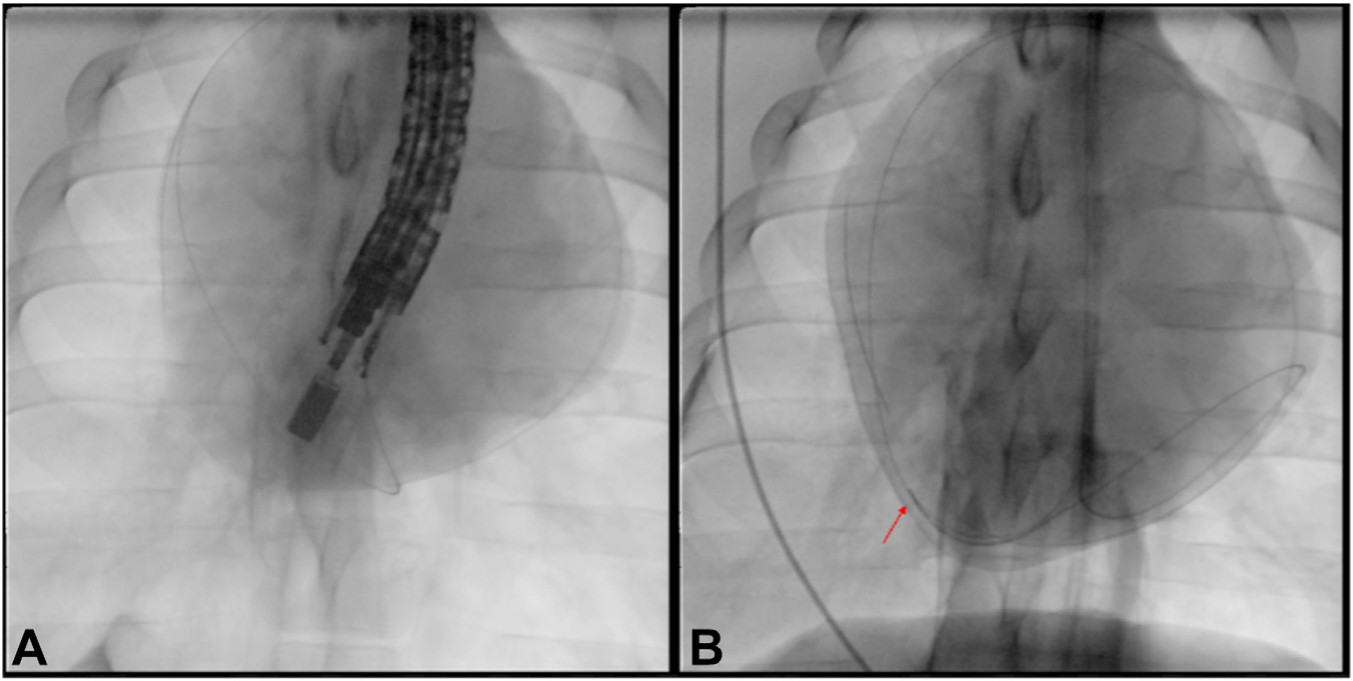
(**A**) Wire in pericardium. (**B**) Catheter placement over wire.

After epicardial access was confirmed, a 0.035-inch Jagwire was passed into the pericardium through the needle and the needle exchanged out. Over the wire, a Jagtome Revolution Rx 3.9F catheter (Boston Scientific) was then passed bluntly over the wire through the esophageal wall, achieving epicardial catheter access (Fig. 8).

**Figure 9:**
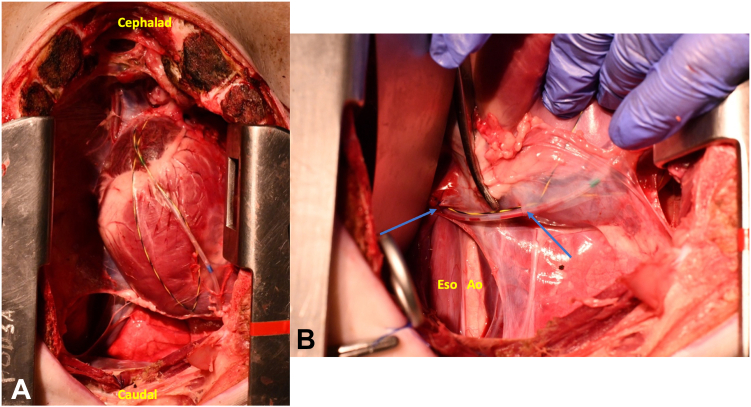
(**A**) Wire and catheter located within the pericardium. (**B**) Wire and catheter passing from the esophagus to the pericardium. *Blue arrow* on the left marks exit site of wire and catheter from esophagus (Eso), past the aorta (Ao) to the *blue arrow* on the right, where the catheter and wire enter the pericardium.

After placement of the catheter, the echoendoscope was exchanged out, the wire and catheter left in place, and the procedure was completed. Total procedure time was approximately 45 minutes via the EUS-guided transesophageal approach. The procedure was terminated and a necropsy performed, confirming proper placement of the catheter in the pericardial space. No epicardial injuries were noted (Fig. 9).

### Hemodynamic stability

The animals maintained oxygen saturation >90% throughout the experiments. Transient arrhythmias were observed in both procedures, resulting in momentary hypotension. These arrhythmias were self-resolving after few ectopic beats, and no animals required cardioversion or vasopressor support. These arrhythmias appeared to be due to transient epicardial irritation during needle manipulation. All animals were stable after resolution of initial ectopy. During the NOTES approach, care was taken to avoid over-insufflation of gas in the mediastinal space, and brief episodes of hypotension were quickly reversed with gas decompression through the endoscope. Vasopressor support was not required.

## Discussion

These experiments show the feasibility of endoscopic epicardial access, as well as NOTES transesophageal mediastinal intervention and pericardial window. Fluoroscopic-guided percutaneous epicardial access is challenging. Adverse events encountered via this approach include cardiac perforation, coronary arterial or venous laceration, laceration of the liver, and pneumothorax. These arise primarily due to the lack of visualization of the pericardium itself. The advantage of an endoscopic or EUS approach is direct visualization of the pericardium to facilitate safer entry. A practical clinical application of this approach is the ability to facilitate subsequent percutaneous pericardial entry after insufflation with carbon dioxide should it be necessary.^9^ We envision a multidisciplinary and cooperative approach with endoscopic epicardial access performed in the electrophysiology suite before cardiac intervention. Each approach has its own advantages and disadvantages, as EUS enables access to the epicardium without having to exit the lumen of the esophagus; it does not, however, have the benefit of a direct sight line for epicardial access that a NOTES dissection enables for added safety. We believe that the most immediately relevant clinical application will be an EUS-guided approach in an adjunctive or assistive role to pericardial intervention.

POEM has been established as a safe and efficacious procedure to treat achalasia and is the prototypical NOTES procedure that has gained widespread clinical adoption. Endoscopists have learned through this experience how to manage the intraluminal and extraluminal space and the unique challenges that can arise. It has been well established that inadvertent access to the mediastinum is common during POEM, resulting in clinically insignificant capnothorax, capnomediastinum, and capnoperitoneum that resolve spontaneously, and clinically insignificant pleural effusion and peritonitis may be experienced without infectious adverse events.^10^ This suggests that traversing the myotomy endoscopically for mediastinal intervention is unlikely to lead to significantly more infectious adverse events than POEM, which is typically performed with only perioperative antibiotics.^11^ However, it remains to be shown whether epicardial access via an endoscopic approach leads to higher rates of infectious adverse events compared with traditional techniques.

The tools used in this experiment are designed pancreaticobiliary and GI luminal use. Although the procedures we described can be accomplished with off-label use of currently available devices, an opportunity exists for development of dedicated endoscopic tools modeled by those used in laparoscopy, video-assisted thoracoscopic surgery, and interventional cardiology to expand the capabilities of surgical endoscopy.

As endoscopists continue to push the limitations of extraluminal endoscopy, the use of flexible endoscopy to address extraluminal organ spaces will grow. Whether through endoscopic or robotic platforms, transluminal surgery increasingly will be explored in the future with additional refinements of techniques and technology. Our study shows both the feasibility of these interventions as well as the opportunity for innovative device development to advance this field. As the breadth of pericardial interventions continues to increase with the development of epicardial ventricular ablation, left atrial appendage ligation, and proactive esophageal protection during left atrial ablation, endoscopic approaches for epicardial access may enable increased safety and applicability to these established procedures as well as the development of new therapeutic and diagnostic strategies for cardiac diseases in the future.

## Disclosure

The following authors disclosed financial relationships: O. Ajijola: Ownership interest in NeuCures Inc. K. Shivkumar: Ownership interest in EP Dynamics Inc; and consultant for Anumana Inc and Nference. E. Dutson: Consultant for Fujifilm. A. Sedarat: Consultant for Boston Scientific, Apollo, Fujifilm, and Steris. All other authors disclosed no financial relationships. This work was made possible by support from National Institutes of Health grants OT2OD023848 (K. Shivkumar and O. Ajijola) and 2T32DK007180-47 (Z.N. Weitzner).

## Supplementary data

Video 1 In this video, we demonstrate directly visualized endoscopic epicardial access and creation of an endoscopic pericardial window, followed by EUS-guided epicardial access in a porcine model.
